# CoVEGI: Cooperative Verification via Externally Generated Invariants

**DOI:** 10.1007/978-3-030-71500-7_6

**Published:** 2021-02-24

**Authors:** Jan Haltermann, Heike Wehrheim

**Affiliations:** 8 Universidad Autónoma de Madrid, Madrid, Spain; 9 University of Twente, Enschede, The Netherlands; Department of Computer Science, Paderborn University, Paderborn, Germany

**Keywords:** Cooperation, Software Verification, Invariant Generation

## Abstract

Software verification has recently made enormous progress due to the development of novel verification methods and the speed-up of supporting technologies like SMT solving. To keep software verification tools up to date with these advances, tool developers keep on integrating newly designed methods into their tools, almost exclusively by re-implementing the method within their own framework. While this allows for a conceptual re-use of methods, it nevertheless requires novel implementations for every new technique.

In this paper, we employ *cooperative verification* in order to avoid re-implementation and enable usage of novel tools as black-box components in verification. Specifically, cooperation is employed for the core ingredient of software verification which is *invariant generation*. Finding an adequate loop invariant is key to the success of a verification run. Our framework named CoVEGI allows a master verification tool to delegate the task of invariant generation to one or several specialized helper invariant generators. Their results are then utilized within the verification run of the master verifier, allowing in particular for crosschecking the validity of the invariant. We experimentally evaluate our framework on an instance with two masters and three different invariant generators using a number of benchmarks from SV-COMP 2020. The experiments show that the use of CoVEGI can increase the number of correctly verified tasks without increasing the used resources.

## 1 Introduction

Recent years have seen a major progress in software verification as for instance witnessed by the annual competition on software verification SV-COMP [2]. This success is on the one hand due to advances in SAT and SMT solving and on the other hand due to novel verification methods like interpolation in model checking [36], automata-based software verification [31] or property directed reachability [16]. Still, automatic verification remains a complex and error-prone task. In particular, it is often the case that one tool can verify a particular class

⋆ This author was partially supported by the German Research Foundation (DFG) under contract WE2290/13-1.

of programs, but fails to verify other classes (or even gives incorrect answers), whereas it is the reverse situation for another tool. Moreover, to keep their tools up to date with novel techniques, tool developers keep on integrating them by re-implementation within their framework.

An approach for changing this unsatisfactory situation is *cooperative verification* (for an overview see [13]). Cooperative verification builds on the idea of letting tools (and thus techniques) cooperate on verification tasks, thereby leveraging the tool's individual strengths. In particular, cooperative verification aims at *black box* combinations of tools, using existing tools off-the-shelf without re-implementation. While this sounds like a natural idea, its realization poses a number of challenges, the major one being the *exchange* and *usage* of analysis information. For cooperation, tools are required to produce (partial) results which other tools can understand and employ in their verification run. With conditional model checking [7], the first proposal of an exchange *format* for verification results was made. A conditional model checker outputs its (potentially partial) result in the form of a *condition* which can be read by other conditional model checkers in order to complete the verification task. Since verification tools normally do not understand conditions, *reducers* [23,9] have been proposed to bring conditions back into a form understandable by verifiers, namely into (residual) programs describing the so far unverified program part. This allows the result of a conditional model checker to be made usable by arbitrary other verifiers. A second type of existing result usage is the *validation* of tool's results [4,34], similar to proof-carrying code [37]. Both of these types are sequential forms of cooperation: a first verifier starts and a second verifier continues, either by completing or by validating a first result.

In this paper, we propose CoVEGI, a cooperation framework which complements these existing approaches by a new type of cooperation. Conceptually, this framework (depicted in Figure 1) consists of a *master verifier* and a number of *helper invariant generators*. The master verifier has the overall control on the verification process and can *delegate* tasks to helpers as well as *continue* its own verification process with (partial) results provided by helpers. The helpers run in parallel as black boxes without cooperation. The task to be delegated is an integral part of software verification, namely *invariant generation*. The framework allows cooperation via outsourcing the task of invariant generation, leveraging the strength of specialized invariant generation tools.

Like for other types of cooperation, the question of the exchange format for results comes up. Here, we have chosen *correctness witnesses* [3] for this purpose. Correctness witnesses are employed in witness validation and certify a verifier's result stating the correctness of a program. These witnesses are particularly well suited for our intended usage, because their format is standardized and a number of verifiers already produce correctness witnesses. To account for the incooperation of helper verifiers not producing witnesses, our framework also foresees the inclusion of *adapters* transforming invariants into correctness witnesses. We provide an implementation of two such adapters. Witnesses are then *injected* into the verification run of the master. For stating the task to be solved by invariant

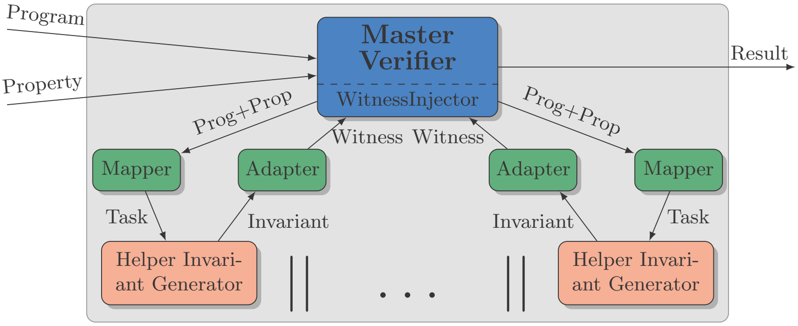

Fig. 1: Cooperative verification via externally generated invariants generators we furthermore require *mappers* transforming program and property to be proven into a task format understandable by the helper tools. Figure 1 depicts our framework for cooperative verification via externally generated invariants. The framework can be arbitrarily configured with different masters and helpers, provided that suitable adapters and mappers are given.

We have implemented CoVEGI within the CPAchecker framework [10] and have employed different configurations of it as master verifier. As helpers we have chosen publicly available verification tools, some producing and one not producing witnesses. We have then experimentally evaluated 14 different combinations of master and helper on benchmarks of the annual competition of software verification SV-COMP [2]. The experiments show an improvement over the verification capabilities of the master tool, without incurring significant overhead. In some cases, the verification time is even decreased in cooperative verification.

Summarizing, we make the following contributions.

- We propose a framework for cooperative software verification based on a master-helper architecture using externally generated invariants.
- We construct 14 different instantiations of the framework using 2 masters and 3 helpers, running both helpers in isolation as well as in parallel.
- For the inclusion of helper verifiers, we implement two adapters, one transforming invariants expressed in the LLVM IR language[1] into correctness witnesses, the other bringing a generated witness into the right format.
- We carry out an extensive experimental evaluation demonstrating the effectiveness and efficiency of collective invariant generation.

## 2 Fundamentals

We aim at the cooperative verification of programs written in GNU C, focusing on the validation of safety properties. To be able to define safety properties, a

[1] https://llvm.org/docs/LangRef.html formal representation of programs as well as their semantics is needed. Thus we briefly introduce the syntax and semantics of programs which we consider here.

We follow the notation of Beyer et al. [6] describing programs as *control-flow automata* (CFAs). A CFA is basically a control-flow graph with edges annotated with program statements. More formally, a program is represented as a control-flow automaton $C = (L, l_0, G)$, consisting of a set of program locations $L$, an initial location $l_0 \in L$ and the control-flow edges $G, G \subseteq L \times Op \times L$. The set $Op$ contains all possible operations on integer variables[2] present in the program, namely conditions (as of conditionals and loops), assignments, method calls and return statements. Figure 2(a) shows a C-program taken from the SV-COMP benchmarks[3], and Figure 2(b) its corresponding CFA. The program also contains a special *error* label, used for encoding the property to be verified. The verification task for this program is to show the non-reachability of the error label at location 9, i.e., for our example program the verifier has to prove that $y$ equals $n$ after the loop which is true (since $n$ is unsigned).

For the semantics, we start by defining program states. Let $Var$ denote the set of all integer variables occurring in programs, $BExp$ the set of boolean expressions and $AExp$ the set of arithmetic expressions over $Var$. Then a *state* $\sigma$ of the program is a mapping from the variables to the integers, i.e., $\sigma : Var \to \mathbb{Z}$. We lift the mapping to also contain the evaluation of arithmetic and boolean expressions so that $\sigma$ maps $AExp$ to $\mathbb{Z}$ and $BExp$ to $\mathbb{B}$. A finite *program path* $\pi$ is a sequence of *transitions* $\langle \sigma_0, l_0 \rangle \stackrel{g_0}{\to} \langle \sigma_1, l_1 \rangle \cdots \stackrel{g_{n-1}}{\to} \langle \sigma_n, l_n \rangle$, such that $\sigma_0$ assigns 0 to all variables, $l_n$ is a leaf in the CFA and $(l_i, g_i, l_{i+1}) \in G$ holds for each transition $\langle \sigma_i, l_i \rangle \stackrel{g_i}{\to} \langle \sigma_{i+1}, l_{i+1} \rangle$ in $\pi$. Infinite program paths are defined analogeously. As for state changes in paths: If $g_i$ is a boolean expression, method call or return statement, then $\sigma_i = \sigma_{i+1}$ holds. If $g_i$ is an assignment $x = a$, where $a \in AExp$, then $\sigma_{i+1} = \sigma_i[x \mapsto \sigma_i(a)]$. Finally, we denote all paths of a program represented by a CFA $C$ by $paths(C)$.

Here, we are interested in verifying safety properties of programs given as CFAs. For the purpose of this paper, we define a *safety property* $P$ as a pair of a location $\ell \in L$ and a boolean condition $\varphi \in BExp$. There can be multiple safety properties required to hold in a program. For our example program of Figure 2 the property is $(8, n = y)$. For the verifier this is encoded in the form

```
8:  if (!(n==y))
9:      Error: return 1;
```

A CFA (or program) $C$ *violates a safety property* $P = (\ell, \varphi)$ when the program reaches location $\ell$ in a state which does not satisfy $\varphi$. More formally, $P$ is violated by $C$, if there is some path $\pi \in paths(C)$, $\pi = \langle \sigma_0, l_0 \rangle \stackrel{g_0}{\to} \langle \sigma_1, l_1 \rangle \cdots \stackrel{g_{n-1}}{\to} \langle \sigma_n, l_n \rangle$ and some $i$, $0 \le i \le n$, such that $\ell_i = \ell$ and $\sigma_i(\varphi) = false$.

[2] In our formalization, we use integer variables only, the implementation covers C programs.

[3] https://github.com/sosy-lab/sv-benchmarks

```c
int main() {
    unsigned int n = nondet();
    unsigned int x = n, y = 0;
    while(x > 0){
        x--;
        y++; }
  // Safety property
    if (!(n == y)) {
        Error: return 1; }
    return 0;}
```

(a) C code example

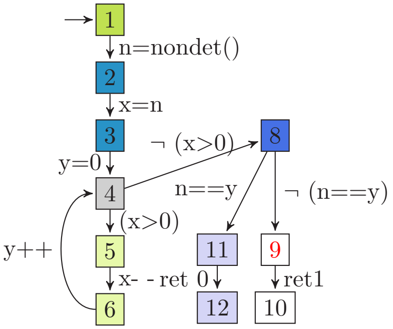

(b) The corresponding CFA

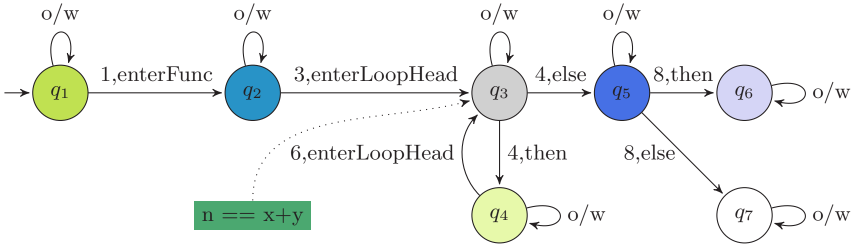

(c) Part of the witness

Fig. 2: An example program, its control flow automaton and one witness

Cooperatively verifying safety of programs is achieved in our framework via external (loop) invariant generation. Syntactically, a *loop invariant* is a boolean expression associated to a loop head. A loop invariant needs to hold (1) before the first loop execution and (2) after each loop execution. The expression $n = x + y$, for instance, is a loop invariant for the program in Figure 2(a), associated to the loop head at location 4. This loop invariant facilitates verification, because in conjunction with the negated loop condition and information about initial variable values it ensures $n$ to be equal to $y$ after the loop. Other valid loop invariants would be $x \geq 0$ or $n = 3 \Rightarrow y \leq 5$, which however all do not help in proving the safety property. Especially the loop invariant *true* does not provide any information. Thus, we call it a *trivial invariant*.

As stated before, we chose *witnesses* (more specifically, correctness witnesses) as exchange format during collective invariant generation. Formally, a witness is a finite state automaton in which transitions are labelled with so called *source code guards* and states can be equipped with boolean expressions. When all these boolean expressions are either *true* or *false*, we call the witness *trivial*. Source code guards are of the form `location,type` where `type` can be `then`, `else`, `enterFunc` and `enterLoopHead`. The guard `o/w` (otherwise) is used if a source code line does not match the other guards present. Via these labels we can match transitions of the automaton with edges in the CFA. Syntactically, correctness witnesses are stored in an XML format and consist of two parts:

(1) general information like the program associated with the witness, and (2) a GraphML representation of the witness automaton. More information and a formal specification of correctness witnesses can be found in [3].

In Figure 2(c), we see a correctness witness for our example program. State $q_3$ is reached by transitions labelled `3,enterLoopHead` or `6,enterLoopHead` and thus corresponds to the loop head at program location 4. Associated with this state is the invariant $n = x + y$.

## 3 Concept

In this section, we introduce our novel concept of **Co**operative **V**erification via **E**xternally **G**enerated **I**nvariants (CoVEGI), shown in Figure 1. The framework contains two sorts of main components: Master verifiers (one) and helper invariant generators (several). Next, we state some requirements on and explain the functionality of these components as well as their cooperation.

### 3.1 Components of the CoVEGI-Framework

The most important component of the framework is the master verifier, which we build out of an existing verifier. The master is responsible for coordinating the verification process and can, if needed, request support from the second type of components, the helpers, in the form of invariants as described by correctness witnesses. Hence, the master is also steering the cooperation.

In the following, we explain the two sorts of main components in more detail:

**Master Verifier** A *master verifier* gets as input the program $C$ as CFA and a safety property $P$. It computes as output a boolean answer $b$, stating whether the property holds, and possibly (but not necessarily) provides an overall witness $\omega$. To be able to process the provided support in form of invariants stored inside of correctness witnesses, a master is required to implement an internal function called *injectWitness*. The function loads a witness, extracts the invariants present in it and injects them into the analysis of the master verifier. The witness injection can either happen before (re-)starting the analysis or during runtime.

**Helper Invariant Generator** A *helper invariant generator* gets as input the program $C$ as CFA and a safety property $P$. It computes as output a set of invariants, stored in a verification witness $\omega'$. The generated invariants are neither required to be helpful for the master verifier nor to be correct. Thus, helper invariant generators are also allowed to generate trivial invariants or invariant candidates which might turn out to be wrong.

We can neither expect existing verification tools which we wish to use as helpers to be able to work on CFAs, nor to understand the safety property or to produce witnesses. Hence, we foresee two further sorts of components in our framework:

Table 1: Overview of the configuration options available

| Name | Description | Values |
|---|---|---|
| `restartMaster` | restart the master after invariant generation | boolean |
| `termAfterFirstInv` | use first witness only | boolean |
| `timerM` | max. time for master until `requestsForHelp` is send | time(s) |
| `timeoutH` | max. time for helpers to generate an invariant | time(s) |

**Mapper** A *mapper* transforms the safety property specification inside the program into the desired input format of the helper. A mapper basically conducts some simple syntactic code replacements. For instance, for our running example some helpers might instead require the safety property to be written as `assert(n==y);` or as `if(!(n==y)) {verifier_error();}`.

**Adapter** An *adapter* generates a correctness witness out of the computed loop invariants of a helper. Furthermore, some helper invariant generators work on intermediate representations (IR) of the C-language (e.g. LLVM) or intermediate verification languages (e.g. Boogie). Then, the computed invariants (formulated in terms of IR-variables) first of all need to be translated back to the namespace of the C-program. An adapter for LLVM is explained in more detail in Section 3.4.

### 3.2 Cooperation within CoVEGI

After having explained the individual components, we define their interaction in the framework. In this paper, we focus on the *parallel* execution of several helpers which implement complementary approaches so that we can leverage their individual strengths. Algorithm 1 describes the form of cooperation. It is steered by several user configurable options which fix aspects like time and resource limits of master and helpers. Table 1 summarizes the configuration options. We next describe them in detail.

**Master options** The following aspects of the master's behavior need to be fixed: First, when to delegate tasks to helpers, and second, how to continue the verification process after invariant generation. For the delegation, we let the master verifier run until it requests support, which can be checked by inspecting the master's flag `requestsForHelp`. The master gets a configurable timelimit (called `timerM`) after which it is expected to send this request. By adding such an explicit request for help, we allow the master to send a request for other reasons (besides the timer) in the future. Then, after invariant generation, the master can either be freshly restarted or continued (option `restartMaster`).

**Helper option** When at least two helpers run in parallel, eventually one of them first computes a witness. We can then either (1) directly stop the other helpers, or (2) wait for all to complete before injecting witnesses into the master. This option is called `termAfterFirstInv`.

**Algorithm 1** CoVEGI-algorithm

```
Input:  C                                                         ▷ CFA
        P                                                         ▷ safety property
        M                                                         ▷ master
        Helpers                                                   ▷ set of helpers
        conf                                                      ▷ configuration
Output: ω                                                         ▷ witness
        b                                                         ▷ result
 1: M.start(C, P, conf.timerM);
 2: wait until (M.requestsForHelp ∨ M.hasSolution());
 3: if (M.hasSolution()) then
 4:     return M.getSolution();
 5: for each H ∈ Helpers do parallel                              ▷ run helpers in parallel
 6:     H.start(C, P, conf.timeoutH);
 7:     wait until (H.timedout() ∨ H.hasSolution() ∨ H.stopped());
 8:     if (H.hasSolution() ∧ nonTrivial(H.getSolution())) then
 9:         witnesses := witnesses ∪ H.getSolution();
10:         if (conf.termAfterFirstInv) then
11:             for each H' ∈ helpers \{ H } do parallel
12:                 H'.stop();                                    ▷ stop other helpers
13: if (M.hasSolution()) then
14:     return M.getSolution();
15: if (witnesses ≠ ∅) then                                       ▷ invariants found
16:     if (conf.restartMaster) then
17:         M.stop();
18:     M.inject(witnesses);                                      ▷ inject witnesses into master
19:     if (conf.restartMaster) then
20:         M.start(C,P, ∞);
21: join(M);                                                      ▷ wait for M to finish
22: return M.getSolution();
```

**Timeouts** Finally, similar to the master, we can set a specific timeout for the helpers which fixes how long they are allowed to try to generate invariants. The timeout option is called `timeoutH`.

Next, we explain the CoVEGI algorithm shown in Algorithm 1 in detail. We assume that master and helpers run as threads and can be started and stopped. We furthermore employ methods `wait` for waiting until some condition is achieved and `join` for waiting for a specific thread to complete.

Initially, the master verifier is started without any helper invariant generators running in parallel (line 1), providing the opportunity to verify programs on its own. It runs standalone until it requests for help (either due to not being able to solve the problem alone or due to hitting its timer) or until it computes a result which is subsequently returned (line 3). Afterwards all helpers are started in parallel (lines 5 and 6). They also run until they reach their timeout, a solution is found or they are stopped. Their solutions (invariants) are inserted into the witness set (line 9). Depending on option `termAfterFirstInv`, either all but the first finished helper are stopped or it is waited until all helpers either computed a solution or ran into their timeout. If invariants (witnesses) have been computed, these are injected into the master (line 18). If the `restartMaster` option is set, the master needs to be stopped before injection and restarted afterwards. Then the master continues and completes its verification (without any further request for help) and the result is finally returned.

*Example 1.* To explain the framework's functionality, we demonstrate the CoVEGI algorithm on the example presented in Figure 2(a). Assume that we instantiate the framework with a master verifier and four helper invariant generators, that are used in parallel[4]. Moreover, we configure the framework as follows: We set `restartMaster` to true, `terminateAfterFirstInv` to false, `timerM` to 50 seconds and `timeoutH` to 300 seconds.

Initially, the master verifier runs standalone and after 50 seconds runtime it requests help. The master runs in parallel with the four helper invariant generators being called. Let us assume that the first helper returns only trivial invariants (after 10s), the second one the invariant $n \geq y$ (after 50s), the third one the invariant $n = x + y$ (after 100s) and the fourth the invariant $n - x - y = 0$ (after 500s). The trivial invariant is ignored (see check in line 8) and when the second helper returns a solution, the third and fourth helper are still not stopped, due to the chosen configuration. The algorithm waits until the third helper computes the invariant and the fourth (only being able to compute an invariant after 500s) hits the timeout after 300s. Then the master is stopped, the invariants $n \geq y$ and $n = x + y$ are injected and the master is restarted. The master verifier can use both invariants and might now compute the correct result.

### 3.3 Witness Injection

As master verifiers need to offer witness injection, we explain a possible procedure for predicate abstraction and k-induction, which are the two techniques we use as masters during the evaluation. For both, the invariants are extracted from the witness and then added to the analysis information already computed by the master verifier. Both analyses store their analysis information in an *abstract reachability graph* (ARG). Broadly speaking, an ARG is a CFA equipped with predicates. More formally, an ARG is a finite state automaton, where nodes, called *abstract states*, consist among others of analysis information (i.e. predicates) and program locations. Two nodes within an ARG are connected if their program locations are connected within the CFA. Note that a program location may occur in multiple abstract states, e.g. when the analysis unrolls a loop. Hence, witness injection has to update all the abstract states for whose program location the witness contains an invariant.

**Predicate Abstraction.** We use a predicate abstraction technique [11], conducting predicate refinement using a CEGAR (counter example guided ab-

[4] In [29] is is shown that more than two helpers does not practically make sense.

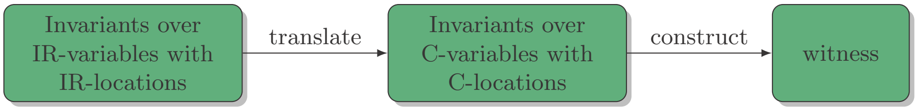

Fig. 3: Workflow of an adapter for an helper working on an IR

straction refinement) scheme [20] with lazy-abstraction [33] and Craig interpolation [32].
*Witness Injection:* The predicate abstraction maintains, for each abstract state, one set of available predicates (called *precision*) and one set of valid predicates. Witness injection is realized by extracting all predicates and the corresponding locations from the invariants. If these predicates contain conjunctions of clauses, these are furthermore split up and inserted individually. Splitting predicates increases the performance due to the fact that SMT solvers perform better on many small predicates than on few larger ones[5]. These predicates are added to the precision of abstract states corresponding to the locations specified in the witness. Thereby, the predicates are used during the next abstraction performed by the analysis. The abstraction function itself guarantees that only predicates from the candidate set being valid at the current location are used. Thus, invalid invariants are ignored. This procedure can also be used when restarting predicate abstraction, by adding the predicates from the witness to the initial precision of the abstract states corresponding to the locations specified in the witness (which is empty otherwise).

**k-Induction**. The basic idea of k-induction [25] is to generalize bounded model checking (BMC) [14] via induction. After proving k-bounded program executions safe using BMC, a generalization is aimed for. Therefore, it generates auxiliary invariants that are continuously refined using a CEGAR based analysis [5]. These invariants are combined with the information generated by BMC and generalized to a safety proof by successfully conducting an induction step.
*Witness Injection:* For both cases, adding invariants into a running analysis or adding before restarting, we make use of the same idea: Whenever a witness is made available to the analysis, the encoded predicates and the program locations are added as candidates to the set of auxiliary invariants, generated by the analysis. New elements in this set are periodically checked for validity by k-induction. Thereby, valid externally generated invariants are conjoined with the predicates stored in the analysis abstract states, corresponding to the invariants location. Invalid invariants are thus ignored.

### 3.4 Adapter for LLVM-based Helper Invariant Generators

Next, we exemplify an adapter for helper invariant generators working on LLVM, following the general construction depicted in Figure 3. Often, tools associates invariants to LLVM basic blocks. A basic block is a code fragment having a single

[5] This has been reported by tool developers and has also shown in our experiments.

entry location (the first) and a single exit location (in general the last location of the block). To construct a witness containing the invariants, we need to translate them and find the matching C-code location for the basic block. For both, we use the LLVM-IR equipped with debug information, using the compiler with launch parameter `-g`. Thereby, we obtain the IR-code fragment of the program in Figure 2(a), shown in simplified form and containing the most important debug information as comments. The example contains two basic blocks, `entry` and `_bb`.

```
entry:
v1 = bitcast i32 (...)* @nondet to i32 ()*        ▷n
v2 = icmp eq i32 v1, 0
br i1 v2, label %error, label %_bb

_bb:
v3 = phi i32 [0, %entry], [v6, %_bb]              ▷y
v4 = phi i32 [v1, %entry], [v5, %_bb]             ▷x
v5 = add i32 v4, -1
v6 = add i32 v3, 1
v7 = icmp eq i32 v5, 0
br i1 v7, label %error, label %_bb          ▷line 4
```

The helper invariant generator computes the invariant $v1 - v4 - v3 = 0$ for the example and associates it with the basic block `_bb`. At first, we need to transform the variables from the IR to C-variables occurring in the program. In this example we can use the debug information, as shown in comments in the code. In general, a more sophisticated procedure is needed since LLVM-IR uses a three address code. Therein, complex expressions are split into several statements using intermediate variables which are resolved to C-expressions.

Afterwards, the transformed invariant needs to be associated with the correct location in the C-code. We analyze the LLVM IR program structure to map the basic blocks back to C-locations. In the example, the block `_bb` is identified as being the loop of the program, thus the invariant is mapped to the loop head. For this, we employed some basic functions provided by PHASAR [41] in our adapter. Finally, we construct the CFA of the C-program, store the invariants at the nodes and convert the equipped CFA to a verification witness.

## 4 Evaluation

In the following, we evaluate different instantiations of CoVEGI. We focus on both effectiveness and efficiency, generally aiming at checking whether the use of CoVEGI can increase the number of correctly solved verification tasks within the same resource limits. A more detailed evaluation of CoVEGI can be found in an extended pre-print [29].

### 4.1 Research Questions

In the evaluation, we were interested in the following three research questions.

Table 2: Summary of tools used as helpers

| Tool | Techniques | Mapper | Adapter |
|---|---|---|---|
| SeaHorn | generation and solving constrained horn clauses | ✘ | ✔ |
| Ultimate-Automizer | predicate abstraction, automata, path-based refinement | ✘ | (✔) |
| VeriAbs | portfolio of 4 different sequential compositions | ✘ | ✘ |

**RQ1.** Can collective invariant generation increase the effectiveness of the master verifier? *Evaluation plan:* We let the framework run with a single invariant generator and compare the results to a standalone run of the master verifier.

**RQ2.** Does cooperation impact the overall efficiency of the verification? *Evaluation plan:* We compare the run time of CoVEGI with one helper against the two master verifiers running standalone.

**RQ3.** Does it pay off to run two invariant generators in parallel? *Evaluation plan:* We let the framework run with two invariant generators and compare the results to a run, where only a single invariant generator is used.

### 4.2 Experimental Setup

**Tools.** To be able to evaluate the performance of our framework CoVEGI, we instantiated it with predicate abstraction and k-induction as master verifiers and three helpers, using existing off-the-shelf invariant generation tools. We based the implementation of our CoVEGI algorithm on CPAchecker[6] 1.9.1. To the best of our knowledge, there are no standalone and publicly available invariant generators, that generate invariants for both, global and local variables, without doing a full verification. To be able to evaluate CoVEGI, we decided to use off-the-shelf verifiers as invariant generators instead, by only using the generated invariants. We thus looked at current and past participants of the annual competition of software verification SV-COMP [2] for invariant generation. We chose the tools SeaHorn [28], UltimateAutomizer [30] and VeriAbs [1]. Both UltimateAutomizer and VeriAbs achieved excellent results in this year's SV-COMP, being the reason to chose them. As third tool we use SeaHorn, a verification tool neither currently participating in the SV-COMP nor producing witnesses. It operates on the LLVM intermediate representation, therefore we used the adapter exemplified in Section 3.4. The three helper invariant generators are used as black-boxes and employ verification techniques complementary to those of both the other helpers and the two masters. An overview of the techniques employed in these tools is given in Table 2. The table also states whether the helpers require mappers and adapters. For VeriAbs and UltimateAutomizer we used the versions as used in the SV-COMP 2020[7]. Due to the fact that there is no precompiled binary of SeaHorn, we employ the docker

[6] https://github.com/sosy-lab/cpachecker, Revision (8646a85)
[7] https://gitlab.com/sosy-lab/sv-comp/archives-2020/tree/master/2020

Table 3: Comparison of the two master verifiers running standalone and using a single helper.

| Tool - Combination | k-induction | | | | predicate abstr. | | | |
|---|---|---|---|---|---|---|---|---|
| | alone | +SH | +UA | +VA | alone | +SH | +UA | +VA |
| correct overall | 146 | 148 | 158 | **163** | 116 | 122 | **132** | 125 |
| correct true | 102 | 104 | 114 | **119** | 78 | 84 | **94** | 87 |
| correct false | 44 | 44 | 44 | 44 | 38 | 38 | 38 | 38 |
| additional true | - | +3 | +13 | **+19** | - | +6 | **+16** | +9 |
| additional false | - | 0 | 0 | 0 | - | 0 | 0 | 0 |
| uniquely solved | 1 | 0 | 8 | 15 | 0 | 0 | 6 | 3 |

container of the latest version[8]. All three helper invariant generators are used in their default configuration.

During evaluation, we used the following default configurations for our own framework: We set `termAfterFirstInv` and `restartMaster` to true, setting the `timerM` to 50s[9] and the `timeoutH` to 300s. In general, we will use the abbreviations SH for SeaHorn, UA for UltimateAutomizer and VA for VeriAbs.

**Verification Tasks.** The verification tasks used are taken from the set of SV-COMP 2020 benchmarks[10]. As we are interested in finding suitable loop invariants, we selected all tasks from the category ReachSafety-Loops. To obtain a more broad distribution of tasks, we randomly selected 55 additional tasks from the categories ProductLines, Recursive, Sequentialized, ECA, Floats and Heap, yielding in total 342 tasks.

**Computing Resources.** We conducted the evaluation on three virtual machines, each having an Intel Xeon E5-2695 v4 CPU with eight cores and a frequency of 2.10 GHz and 16GB memory, running an Ubuntu 18.04 LTS with Linux Kernel 4.15. We run our experiments using the same setting as in the SV-COMP, giving each task 15 minutes of CPU-time on 8 cores and 15GB of memory. We employed Benchexec guaranteeing these resource-limitations [12].

**Availability.** Our tool and all experimental data are available[11].

### 4.3 Experimental Results

We implemented the CoVEGI-framework as proof-of-concept in the CPAchecker-framework. For this, we had to extend the existing implementations of k-induction and predicate abstraction with witness injection. For the helper invariant generators we did not change a single line of code, only adding adapters if needed. Integrating helpers like VeriAbs, not requiring an adapter or a mapper, can be done within a few lines of code. Although the implementation is a proof-of-concept, this shows that the presented framework works in practice

[8] suggested by the developers; used docker seahorn/seahorn-llvm5 (4c01c1d)
[9] Which has turned out to be a preferable value, as we explain in [29]
[10] https://github.com/sosy-lab/sv-benchmarks/releases/tag/svcomp20
[11] https://covercig.github.io/covegi/

and is applicable to all kinds of off-the-shelf helper invariant generators, those producing verification witnesses as well as those generating invariants in IR.

**RQ1 (Effectiveness).** To evaluate whether a master verifier benefits from the support of a helper, we execute a combination of a master and a helper in the default configuration and compare it to the master running standalone. Here, we are interested in the number of *correct* verification results, i.e., the verifier correctly reporting the safety property to be fulfilled (result *true*) or not (result *false*). Running standalone, k-induction can correctly solve 146 of the verification tasks, predicate abstraction 116.

Table 3 gives the results of this experiment. In the table we see the overall number of correct results, the number of correct *true* and correct *false* results plus the number of tasks additionally solved when using a helper and uniquely solved by the configuration. Through the cooperative invariant generation, the performance of both masters is increased. As expected, this applies to verification tasks with fulfilled safety property only, i.e., the invariant generators can help in proving a property to hold, but cannot help in refuting properties (as they correctly do not generate invariants in these cases). Besides the additionally solved tasks, there is also one (for SH and UA) and two (for VA) tasks, respectively, which cannot be correctly solved anymore. In these cases, the master consumes most of the CPU time available, hence sharing resources in cooperation with the helpers results in a timeout.

On our data set, the total number of correctly solved tasks using CoVEGI increases by 12% for k-induction and 14% for predicate abstraction as master.

**RQ2 (Efficiency).** Next, we evaluate the efficiency of CoVEGI, analyzing the CPU time spend solving the verification tasks. As CoVEGI eventually shares the CPU time between master and helpers, we expect that more time is needed to compute a correct result after the helper is started.

Figure 4 shows two quantile plots of the verification runs, 4(a) with k-induction and 4(b) with predicate abstraction as master. A datapoint $(x, y)$ in the plot means that the verifier computes the $x$-fastest correct results in at most $y$ seconds. As CoVEGI instances behave like masters standalone in the first 50 seconds, we only show results *not* solved within these 50 seconds. We see that for tasks requiring a low amount of time, all instances (including the master alone) require a similar amount of CPU time. For tasks requiring more time, CoVEGI is actually often faster, the extreme being predicate abstraction as master which alone is unable to solve more difficult tasks in the given time.

We exemplarily also compared the CPU time of k-induction standalone with CoVEGI using VeriAbs as helper *per task*. It turns out that sharing does only slightly impact the runtime, as shown in Figure 5. The scatter plot compares the CPU time of k-induction standalone as master and k-induction supported by VeriAbs, in case both tools solved the task correctly. A datapoint $(x, y)$ means that k-induction standalone takes $x$ seconds to solve the task and in combination with VeriAbs $y$ seconds. The red dashed box contains all tasks solved within 50 seconds, where both tools behave equally, since the master does not request for

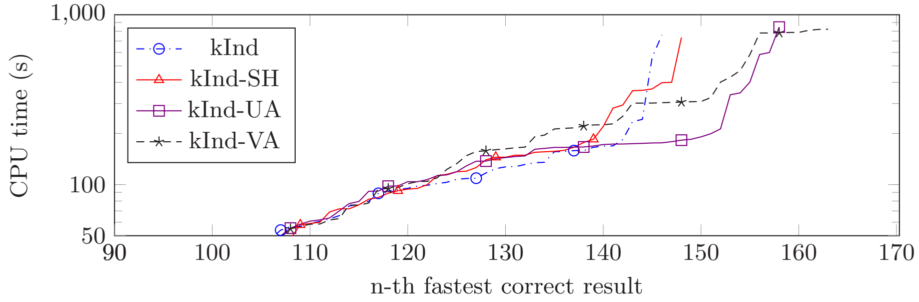

(a) CoVEGI using k-induction as master

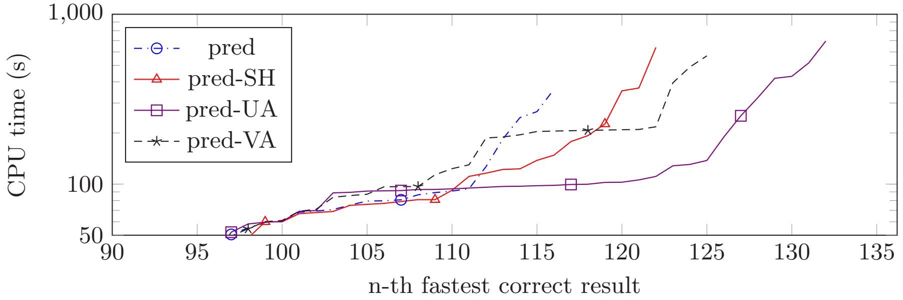

(b) CoVEGI using predicate abstraction as master

Fig. 4: Quantile plots for CoVEGI using different single helpers.

help in these cases. We see some tasks for which helping increased the runtime, but also some for which it decreased it. In most of the cases, the CPU time used by CoVEGI is not significantly higher.

Finally, we compare the average CPU time needed to correctly solve a task. Table 4 shows the average time needed for all tasks and – in brackets – for the correctly solved tasks only. We observe that the runtime increases when only looking at correctly solved tasks (in particular for VeriAbs), however, when considering all tasks the CPU time is even decreased. The latter effect is due to the number of timeouts of the master decreasing when cooperating with helpers. Concluding, we can make the following observation.

> On our dataset, collaborative invariant generation does not negatively impact the effectiveness; in some cases we even see small improvements.

**RQ3 (Combination of helpers).** In RQ3, we were interested in finding out (a) whether it is beneficial to run two invariant generators in parallel, and (b) if yes, which pair is best for this. We thus studied the number of correctly solved tasks using the three possible pairs of helpers, each running two helpers in parallel. Table 5 shows the results.

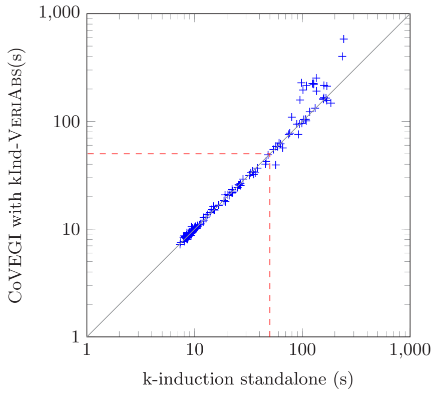

Fig. 5: Scatter plot for kInd and kInd-VA

Table 4: Total CPU time for all tasks and average CPU time taken for a correct answer in brackets, both in seconds.

| **Master** | **kInd** | **Pred** |
|---|---|---|
| standalone | 491.000 (50) | 479.000 (30) |
| +SH | 489.000 (63) | 468.000 (39) |
| +UA | 477.000 (68) | 454.000 (51) |
| +VA | 482.000 (107) | 470.000 (49) |

Table 5: Number of correctly solved tasks using different forms of cooperation with two or three helpers running in parallel.

| **Master** | +SH-UA | +SH-VA | +UA-VA | +SH-UA-VA |
|---|---|---|---|---|
| **k-induction** | 153 | 156 | **163** | 154 |
| **predicate abstr.** | 130 | 130 | **136** | 129 |

For checking whether parallel execution of helpers is beneficial, these numbers need to be compared against those for a single helper as given in Table 3. We see that predicate abstraction benefits from using two helpers, especially using UltimateAutomizer and VeriAbs. Using CoVEGI with these tools perfectly combines their strengths, thereby increasing the number of correctly solved tasks in total by 17%. In contrast, it turns out that for k-induction none of the combinations of two helpers outperforms CoVEGI using VeriAbs only. For UltimateAutomizer and VeriAbs as helpers, the total number does not change, only the set of solved tasks. For instance, nearly 50% of the additional tasks solved by kInd-UA-VA are not solved using kInd-UA and vice versa. This result is based on the fact that they have to share the available CPU time in the combination. Hence, tasks that are solved using one of them as helper alone could not be solved anymore in a combination because of timeouts. This phenomenon is even more an issue when running all three helpers in parallel.
The combination of all three helpers solves only 154 tasks correctly for k-induction and 129 for predicate abstraction. In addition, we evaluated different values for parameter `timeoutH` in [29], whereas it turns out that waiting for all helpers to finish does not increase the number of correctly solved tasks.

On our dataset, CoVEGI can increase the total number of correctly solved tasks using UA and VA in parallel; in general waiting for the other tool to also finish its computation does not pay off.

### 4.4 Threads to Validity

We have conducted our evaluation using a random sample of tasks as well as those in the category Loops. Although this guarantees some diversity, our findings may not completely carry over to arbitrary real-world programs.

The experiments are conducted using the reliable framework Benchexec on identical machines with same resource limitations, guaranteeing comparable results. As SeaHorn is used within a docker-container, its CPU usage however cannot be measured by Benchexec. We therefore measured this externally, rounded it up and added it to the measured CPU time, obtaining a lower bound for the correctly solved tasks. Thereby, all results stay valid, especially of the best performing instantiations of CoVEGI, as they do not use SeaHorn.

Our implementation of CoVEGI relies on the correctness of the used master verifiers and helpers (which are given) as well as on the adapters (which we build). An incorrectly translated invariant may however influence the performance only negatively. Both master verifiers used as well as UltimateAutomizer and VeriAbs are participating in the annual SV-COMP, hence they might be tuned to the tasks employed. This does however not influence the validity of the results since our interest is in the *additional* number of tasks solved by cooperation, not the solved ones per se.

## 5 Related work

In this paper, we presented a framework for cooperative verification via collective invariant generation. The idea of collaboration for verification by combining known techniques has been widely employed before. For instance, there are combinations of verification with testing approaches [21,22,26,18,19,24] and with approaches for invariant generation [40,27,39,15,17]. The latter combinations are conducted in a *white box* manner using strong coupling between the components, making the addition of a new approach a challenging task. Our framework conceptually decouples the invariant generation from the verification, making it more flexible. In addition, using a black box integration with defined exchange formats allows us to easily exchange or integrate new approaches.

There are also existing concepts for collaboration between different techniques in a *black-box* manner. Conditional model checking is a technique for sequentially composing different model checkers, sharing information between the tools in form of conditions [7]. Beyer and Jakobs developed a concept for combining model checking with testing [8]. Although both approaches enable cooperation, none combines a verification tool and tools for invariant generation.

We next shortly discuss three approaches which are conceptually closer to our framework. Frama-C is a framework for code analysis, aiming for analyzing industrial size code [35]. The framework contains different plugins, each implementing a verification or testing technique. The plugins can exchange information in form of ASCL source code annotations. Within Frama-C, the analyzers can collaborate by being either sequentially or parallelly composed. For this, partial results produced by an analysis can be completed by a second one or several partial results computed in parallel are composed to a complete result. Frama-C offers the general possibility to define cooperation between existing plugins. To the best of our knowledge, Frama-C does however not provide a conceptual collaboration of a verification approach and tools for invariant generation driven by the verification approach's demand for support.

The approach of using continuously refined invariants for k-induction [5] uses a lightweight dataflow analysis which can be considered to be a helper for verification. Therein, the supporting invariant generator runs in parallel to the k-induction analysis. Compared to our framework, the main difference is the form of cooperation used. Beyer et al. use a white-box integration for the cooperation between k-induction and the invariant generator, building hardly wired connections between both analyses and sharing the information *inside* the tool. Thus, integrating external tools is hard to achieve. Moreover, the approach is designed to work for k-induction only. Note that an analogeous approach is proposed by Brain et al. [17].

Pauck and Wehrheim proposed CoDiDroid, a framework for cooperative taint flow analysis for Android apps [38]. Within their framework, different analysis tools with specialized capabilities are combined as black-boxes. CoDiDroid is however tailored to the needs of Android taint flow analysis, thus the exchanged information differs. Thus CoDiDroid is not able to orchestrate or exchange information on safety analysis with shared invariant generation.

To summarize, there are a lot of existing approaches for cooperative verification, but most of them are white-box combinations, and the existing black-box combinations are not general enough to allow for collective invariant generation.

## 6 Conclusion

In this paper, we have presented a novel form of black box cooperation for software verification via externally generated invariants. Within the configurable framework named CoVEGI, the so called master verifier steering the verification process is able to delegate the task of invariant generation to one or several helper invariant generators.

We implemented CoVEGI within the CPAchecker framework using k-induction and predicate abstraction as master analysis supported by three existing helpers SeaHorn, UltimateAutomizer and VeriAbs. Our evaluation on a set of SV-COMP verification tasks shows that CoVEGI increases the number of correctly solved tasks without increasing the overall verification time. The best combination of helpers, UltimateAutomizer and VeriAbs in parallel, yields an increase of 12% for k-induction and 17% for predicate abstraction.

Next, we plan to enhance the cooperation by analyzing the behavior of the master in order to identify an optimal point to request for help. Moreover, extending CoVEGI by additionally taking error traces found by the helper into account is also scheduled. In addition, we intend to investigate whether a selection of helpers on the basis of the given verification task is beneficial.
